# A Single nucleotide polymorphism in the *ALDH2* gene modifies the risk of esophageal squamous cell carcinoma in *BRCA2* p.K3326* carriers

**DOI:** 10.1371/journal.pone.0292611

**Published:** 2023-11-09

**Authors:** Neda Zamani, Agata Szymiczek, Ramin Shakeri, Hossein Poustchi, Akram Pourshams, Steven Narod, Reza Malekzadeh, Mohammad R. Akbari

**Affiliations:** 1 Women’s College Research Institute, University of Toronto, Toronto, Canada; 2 Faculty of Medicine, Institite of Medical Science, University of Toronto, Toronto, Canada; 3 Digestive Disease Research Institute, Tehran University of Medical Science, Tehran, Iran; 4 Dalla Lana School of Public Health, University of Toronto, Toronto, Canada; Neyshabur University of Medical Sciences, ISLAMIC REPUBLIC OF IRAN

## Abstract

Esophageal squamous cell carcinoma (ESCC) has a very high incidence rate in northeastern Iran. Our team previously reported the BReast CAncer gene 2 (*BRCA2*) p.K3326* mutation as a moderately penetrant ESCC susceptibility variant in northern Iran (odds ratio (OR) = 3.64, 95% confidence interval (CI) = 1.74–7.59, *P* = 0.0003). Recently, it has been reported that aldehydes can induce BRCA2 haploinsufficiency in cells with a heterozygous pathogenic *BRCA2* mutation and predispose them to carcinogenic effects. Based on this observation, we speculate that dysfunctional variants in Aldehyde Dehydrogenase 2 Family Member (*ALDH2*) may result in aldehyde-induced BRCA2 haploinsufficiency and increase cancer risk in *BRCA2* mutation carriers. In support of this hypothesis, our team recently reported the breast cancer risk modifying effect of an *ALDH2* common polymorphism, rs10744777, among Polish carriers of the *BRCA2* p.K3326* mutation. In the current case-control study, we aimed to investigate the ESCC risk modifying effect of this *ALDH2* polymorphism among *BRCA2* p.K3326* mutation carriers. We assessed the interaction between the *ALDH2* rs10744777 polymorphism and *BRCA2* p.K3326* mutation in ESCC risk by genotyping this *ALDH2* variant in the germline DNA of 746 ESCC cases and 1,373 controls from northern Iran who were previously genotyped for the *BRCA2* p.K3326* mutation. Among a total of 464 individuals with TT genotype of the *ALDH2* rs10744777 polymorphism, which is associated with lower *ALDH2* expression, we found 9 of 164 cases versus 3 of 300 controls who carried the *BRCA2* p.K3326* variant (OR = 5.66, 95% CI = 1.22–26.2, *P* = 0.018). This finding supports our hypothesis that the *ALDH2*-rs10744777 TT genotype may be a significant risk modifier of ESCC in individuals with a *BRCA2* p.K3326* mutation.

## Introduction

After cardiovascular diseases, cancer is the second major cause of mortalities worldwide. It has been estimated that the global incidence of cancer will surpass 25 million by 2032. The etiology of cancer involves a complex interplay among genetics and environmental factors. The adverse impact of cancer on affected individuals’ lifespan and the economic burden it imposes on both patients and societies signify the importance of exploring new diagnostic and predictive markers [1,2].

Among different cancer types, esophageal cancers remain a major global health concern, with 604,100 new cases and 544,076 deaths in 2020 [3]. There is a considerable geographical variation in the incidence of esophageal cancers, and very high incidence rates are observed in a geographical stretch known as the Central Asian Esophageal Cancer Belt [3,4]. A part of this belt is a region in northeastern Iran, settled mainly by Turkmen. The age-standardized incidence rate of esophageal cancers is 13.0 per 100,000 population in this region, with the majority being classified as ESCCs [5]. This incidence rate of ESCCs is among the highest in the world [5,6].

The most reported risk factors for ESCC are environmental factors such as smoking, alcohol consumption, thermal injuries, and low socioeconomic status [7–9]. However, some studies have reported a strong familial component to ESCC, implying that genetic background plays a significant role in the development and high incidence rates of esophageal cancer, especially in high-risk populations [10–13]. Among genetic factors, the contribution of *BRCA2* mutations in the etiology of ESCCs has been observed among high-risk Chinese populations [12–14], Turkmen population of Iran [15], and a high-risk population in India [16].

*BRCA2*, which is a tumor suppressor gene, was initially reported as a breast cancer susceptibility gene in 1995 [17]. Further research revealed the role of *BRCA2* germline mutations in developing ovarian, prostate, and pancreatic cancers [18–20]. Unlike most *BRCA2* truncating variants that confer a high risk of breast and ovarian cancers [21], the *BRCA2* p.K3326* mutation is regarded as a low-penetrant susceptibility allele for these hormone-related cancers [22–24]. However, this mutation has shown to be a highly to moderately penetrant mutation in other cancer types, including pancreatic cancer, squamous cell carcinomas of the lung, upper aerodigestive tract, and esophagus [25–28]. By studying the population in northern Iran, where there is a considerable risk of developing ESCC, our team previously reported the *BRCA2* p.K3326* mutation in 27 of 746 ESCC cases and in 16 of 1,373 controls (OR = 3.64, 95% CI = 1.74–7.59, *P* = 0.0003) [26].

Recently, a novel carcinogenicity mechanism in individuals with a heterozygous *BRCA2* truncating mutation has been proposed by Tan et al. [29]. They observed that aldehydes, including formaldehyde and acetaldehyde, can induce BRCA2 haploinsufficiency by triggering proteasomal degradation of the BRCA2 wild-type proteins in cells heterozygous for a pathogenic *BRCA2* truncating mutation and predispose them to DNA repair anomalies, genomic instability, and possibly cancer [29].

Based on this observation, it is plausible that mutations in *ALDH2*, a gene coding for aldehyde dehydrogenase to detoxify acetaldehyde, may elevate cancer risk in individuals with a heterozygous *BRCA2* truncating mutation such as the *BRCA2* p.K3326* mutation. To evaluate this hypothesis, our team recently investigated the association of the *BRCA2* p.K3326* mutation with breast cancer risk in the context of an *ALDH2* intronic variant (rs10744777) among 11,873 breast cancer patients and 7,615 ethnically matched controls from Poland [30]. The *ALDH2* rs10744777 is an intronic variant located at position chr12: 111795214 (GRCh38.p14) and is highly polymorphic among human population. Although the C allele is the major allele among Asians with allele frequency of 91.2%, the T allele is the major allele among Europeans with an allele frequency of 67.8%. This variant has been reported in association with ischemic stroke risk [31]. The effect of this variant on the ALDH2 protein is poorly understood, but it has been regarded as an expression quantitative trait locus (eQTL) for ALDH2 in monocytes, with the T allele associated with a lower *ALDH2* expression compared to the C allele (β = 0 .159, P value = 8.75 E-11) [32]. Among those who were homozygous for the *ALDH2*-rs10744777 T allele, the OR for developing breast cancer associated with the *BRCA2* p.K3326* mutation was 1.72 (95% CI: 1.19–2.48, P = 0.003). While among those with CC/CT genotypes of the *ALDH2* rs10744777, the carriers of the *BRCA2* p.K3326* mutation did not have a higher risk of breast cancer compared to non-carriers (OR = 1.05, 95% CI: 0.73–1.51, P = 0.81) [30]. Our results suggest the breast cancer risk-modifying effect of the *ALDH2*-rs10744777 TT genotype among carriers of the *BRCA2* p.K3326* mutation.

Having observed this interaction in breast cancer, we aimed to investigate the association of the *BRCA2* p.K3326* mutation with ESCC risk in the context of the *ALDH2* rs10744777 variant. For this purpose, we genotyped the *ALDH2* rs10744777 variant among 746 ESCC cases and 1,373 controls from northern Iran who were previously genotyped for the *BRCA2* p.K3326* mutation [26].

## Materials and methods

### 1. Study subjects

This study is a part of investigations into the etiology of upper gastrointestinal cancers in northern Iran [33]. Study subjects were from cities of Gonbad, the second largest city in Golestan province in northeastern Iran, and Ardabil, the largest city in Ardabil province in northwestern Iran, with high and intermediate rates of ESCC, respectively [34,35]. The majority of the population in Golestan province are Turkmen, and the rest of the population consists of Persians, Turks, Sistanies, Balouches, and Kurds. Nearly all residents of Ardabil province are Turks. Cases and controls were recruited from August 2001 to May 2008. Verification of ESCC diagnosis for all cases was done by upper gastrointestinal endoscopy and subsequent evaluation of tumour biopsies [36]. Written informed consent was obtained from each participant. The data of the participants was handled confidentially. DNA samples were kept anonymous for investigators and the results were not linked to participants’ identities in the dataset. The Ethics Boards of the Digestive Disease Research Institute (DDRI) of Tehran University of Medical Sciences and the Women’s College Hospital approved the study protocol.

#### 1.1. Cases

Overall, 746 cases were recruited for the study, including 281 Turkmen and 465 non-Turkmen ESCC patients. The non-Turkmen were comprised of Turks (n = 304), Persians (n = 114), Sistanies (n = 28), Kurds (n = 10), and Balouches (n = 9). Ethnicity was defined as Turkmen, Turk, Persian, Sistani, Balouch, or Kurd based on pedigree information of four consecutive generations or self-reports if pedigree data was not available. The mean age at the time of diagnosis was 63.6 years, and 50.9% of the study cases were male (n = 380). Other demographic information included smoking status, opium and alcohol consumption, and a family history of cancer.

#### 1.2. Controls

Overall, 1,373 controls were registered in the study, including 811 Turkmen and 562 non-Turkmen. The non-Turkmen consisted of Persians (n = 133), Turks (n = 322), Sistanies (n = 79), Balouches (n = 19), and Kurds (n = 9). Control subjects were hospital patients with a health problem other than cancer (n = 898) or healthy individuals taken from the Golestan Cohort Study (GCS) in northeastern Iran (n = 475) [34]. No controls had a personal history of any type of cancer. The mean age for controls was 55.2 years, and 51.0% of them were male (n = 700). Table 1 summarizes the demographic features of 746 cases and 1,373 controls in our study.

**Table 1:** Demographic characteristics of the ESCC cases and controls.

Variable	Cases	Controls	*P* value
Total number (n)	746	1,373	-
Age, mean (range)	63.6 (25–89)	55.2 (24–90)	< 0.001
Gender, n (%)			
Male	380 (50.9)	700 (51.0)	1.0
Female	366 (49.1)	673 (49.0)	
Ethnicity, n (%)			
Turkmen	281 (37.7)	811 (59.0)	< 0.001
Turk	304 (40.7)	322 (23.5)	
Persian	114 (15.3)	133 (9.7)	
Sistani	28 (3.7)	79 (5.8)	
Balouch	9 (1.2)	19 (1.4)	
Kurd	10 (1.3)	9 (0.6)	
Ever smoker, n (%)			
Yes	200 (27.9)	339 (25.4)	0.14
No	516 (72.1)	1,018 (75.0)	
Ever opium user, n (%)			
Yes	158 (29.1)	239 (20.2)	< 0.001
No	382 (70.9)	943 (79.8)	
Ever alcohol drinker, n (%)			
Yes	37 (5.0)	57 (4.2)	0.22
No	701 (95.0)	1,307 (95.8)	

### 2. Variants genotyping

The *BRCA2* p.K3326* variant was previously genotyped among all studied subjects [26]. Briefly, Germline DNA was extracted from peripheral blood lymphocytes. iPLEX chemistry was applied on a MALDI-TOF MassARRAY system (Sequenom Inc., San Diego, CA, USA) to genotype the p.K3326* variant among Turkmen studied subjects, which included 281 ESCC cases and 811 controls. The procedure was conducted in compliance with the manufacturer’s standard protocol. Cases and controls were distributed evenly in the DNA plates. A total of 190 samples with previously identified sequences were regarded as the quality control group, and their genotype calls from the MassARRAY genotyping were in 100% concordance with their sequencing results. The average genotyping call rate was 100%. Genotyping of the non-Turkmen studied subjects, which included 465 ESCC cases and 562 controls, was performed by using the TaqMan genotyping assay (*BRCA2* p.K3326* assay ID: C___27537307_20) on ABI 7500 fast real-time system (Applied Biosystems Co., Foster City, CA, USA). There were 10% blinded duplicate samples in each plate; the mean concordance rate was 100%. The average genotyping call rate was 100%.

For the purpose of our study, we further genotyped the *ALDH2* rs10744777 among all study subjects by applying the TaqMan genotyping assay (*ALDH2* rs10744777 assay ID: C___2548076_10) on ABI 7500 fast real-time system (Thermo Fisher Scientific, Waltham, MA, USA). There were 10% blinded duplicate samples in each plate; the mean concordance rate was 100%. The average genotyping call rate was over 98%.

### 3. Data analysis

The permutation version of the exact test was done to test for Hardy-Weinberg Equilibrium. Fisher’s exact test was applied to compare genotype frequencies between case and control subjects. Genotype comparisons were made under the dominant and recessive models. The significance level of α = 0.05 was used for all comparisons. We applied a multivariate logistic regression model to calculate adjusted ORs. Covariates included age, gender, ethnicity, smoking, alcohol drinking and opium use. Ethnicity was defined as Turkmen, Turk, Persian, Sistani, Balouch, or Kurd. All analyses were done by SNP & Variation Suite 8 (Golden Helix Inc., Bozeman, MT, USA).

## Results

We studied 746 ESCC patients, including 380 (50.9%) males and 366 (49.1%) females, with the mean age of 63.6 years old (ranging from 25 to 89 years) at the time of diagnosis. The control group was composed of 1,373 individuals, including 700 (51.0%) males and 673 (49.0%) females, with the mean age of 55.2 years old (ranging from 24 to 90 years). A total of 898 control subjects were hospitalized patients with a diagnosis other than cancer, and the remaining controls included 475 healthy individuals who were enrolled in the GCS in northeastern Iran [34]. Table 1 summarizes the demographic characteristics of the studied case and control subjects.

Among studied subjects, 464 individuals were homozygous for the *ALDH2* rs10744777 T allele, which is associated with a lower expression level of the *ALDH2*, 1,031 individuals were heterozygous, and 588 individuals were homozygous for the C allele. This variant was not associated with the ESCC risk itself (Table 2). Of the total 464 individuals who had TT genotype of the *ALDH2* rs10744777, the frequency of the *BRCA2* p.K3326* variant was 5.49% (9 out of 164) among ESCC cases and 1.00% (3 out of 300) among controls (OR = 5.75, 95% CI = 1.53–21.5, *P* = 0.005). After adjusting for age, gender, ethnicity, smoking, alcohol drinking and opium use, *BRCA2* p.K3326* carriers had a 5.66-fold elevated risk of developing ESCC compared to non-carriers with TT genotype of the *ALDH2* rs10744777 (OR = 5.66, 95% CI = 1.22–26.2, *P* = 0.018) (Table 3).

**Table 2:** Frequency of *ALDH2* rs10744777 variant among ESCC patients and controls.

Genotype	Cases N (%)	Controls N (%)	OR (95% CI)	*P* Value	OR (95% CI)^a^	*P* Value^a^
CC	194 (26.0)	394 (28.7)	Ref.	NA	Ref.	NA
CT	377 (50.5)	654 (47.6)	1.17 (0.95–1.45)	0.16	1.25 (0.96–1.62)	0.10
TT	164 (22.0)	300 (21.8)	1.11 (0.86–1.43)	0.43	1.11 (0.80–1.54)	0.52
Dominant			1.15 (0.94–1.41)	0.18	1.20 (0.94–1.55)	0.14
Recessive			1.00 (0.84–1.25)	1.00	0.96 (0.73–1.26)	0.76

^a^ Adjusted for age, gender, ethnicity, smoking, alcohol drinking and opium use employing logistic regression analysis.

**Table 3:** *BRCA2* p.K3326* frequency according to the genotypes of the *ALDH2* rs10744777 variant.

	*BRCA2* p.K3326* frequency					
*ALDH2* rs10744777	Cases (746)	Controls (1,373)	OR (95% CI)	*P* value	OR (95% CI)^a^	*P* value^a^
TT	9/164 (5.49%)	3/300 (1.00%)	5.75 (1.53–21.5)	0.005	5.66 (1.22–26.2)	0.018
CC+CT	18/571 (3.15%)	13/1,048 (1.24%)	2.59 (1.26–5.33)	0.012	3.12 (1.34–7.28)	0.008
CT+TT	21/541 (3.88%)	12/954 (1.26%)	3.17 (1.55–6.50)	0.001	3.80 (1.65–8.76)	0.001
CC+CT+TT	27/746 (3.62%)	16/1,373 (1.17%)	3.18 (1.70–5.95)	0.0003	3.64 (1.74–7.59)	0.0004

^a^ Adjusted for age, gender, ethnicity, smoking, alcohol drinking and opium use employing logistic regression analysis.

## Discussion

The significant role of the *BRCA2* p.K3326* in susceptibility to ESCC among the studied population was previously reported by our team (OR = 3.64, 95% CI = 1.74–7.59, *P* = 0.0004) [26]. Here, we showed that among individuals who were homozygous for the *ALDH2*-rs10744777 T allele, which is associated with a lower expression of *ALDH2*, the *BRCA2* p.K3326* mutation conferred a much higher risk of ESCC (OR = 5.66. 95% CI = 1.22–26.2, *P* = 0.018).

The *BRCA2* p.K3326* mutation results from the substitution of adenine by thymidine at nucleotide 9,976 in exon 27 of the *BRCA2* gene (NM_00059.3), which leads to the loss of the final 93 C-terminus amino acids of the BRCA2 protein. This stop-gain variant was first reported by Mazoyer et al. as a benign variant which is not associated with the risk of breast or ovarian cancer [37]. Later, several studies with considerably larger sample sizes re-evaluated the role of p.K3326* mutation in developing these cancers, and reported a modest but statistically significant increased risk of breast and ovarian cancers associated with carrying this mutation [22–24]. Using data from the large iCOGS study, Meeks et al. reported the p.K3326* variant among 852/41,081 breast cancer patients and 637/38,693 controls (OR = 1.28, 95% CI = 1.17–1.40), and also among 322/14,514 invasive ovarian cancer patients and 411/23,111 controls (OR = 1.26, 95% CI = 1.10–1.43) [22]. Unlike the small effect on the risk of breast and ovarian cancers, the *BRCA2* p.K3326 mutation has shown to be associated with a remarkably increased risk of several other cancers. A recent genome-wide association study, which included 21,594 lung cancer patients and 54,156 control subjects, showed a 2.47-fold increased risk of lung squamous cell carcinoma among p.K3326* carriers (OR = 2.47, 95% CI = 2.03–3.00) [25]. A positive association between *BRCA2* p.K3326* mutation and the risk of developing pancreatic cancer has been reported among both familial (8/144 cases versus 3/250 controls, OR = 4.84, 95% CI = 1.27–18.55) and sporadic (69/2,835 cases versus 73/5,446 controls, OR = 1.78, 95% CI = 1.26–2.52) pancreatic ductal adenocarcinomas [28,38]. More recently, Delahaye-Sourdeix et al. reported the p.K3326* mutation in 149/5,942 upper aerodigestive tract squamous cell carcinoma patients and 75/8,086 controls (OR = 2.53, 95% CI = 1.89–3.38) [27]. Considering the anatomical site of cancer, this group reported a 3.30-fold increased risk of ESCC among p.K3326* carriers which revalidated the previous report by our team that observed a significant association of p.K3326* mutation with ESCC risk among our studied population (OR = 3.64, 95% CI = 1.74–7.59, *P* = 0.0004) [26].

The C-terminus of the BRCA2 protein contains a binding domain essential for the co-localization of RAD51 and monoubiquitinated FANCD2, crucial enzymes for guarding genomic stability through homologous recombination repair (HRR) of double-stranded DNA breaks. Cells missing the *BRCA2* coding region of exon 27 have shown impaired co-localization of BRCA2, RAD51, and FANCD2 complex onto the DNA [39,40]. Studies on mice with deletions of exon 27 reported increased susceptibility to various cancers [41]. Another critical role of the BRCA2-RAD51 complex in maintaining genomic integrity is a replication-specific mechanism which is distinct from repair via HRR. This recently proposed function involves preventing the degradation of nascent strands at stalled replication forks by MRE11, a nuclear protein responsible for this fork instability. This function of the BRCA2-RAD51 complex appears to be important in guarding genomic stability and HRR of double-stranded breaks [42]. The truncation of the BRCA2 C-terminus might interfere with the formation of BRCA2-RAD51 complex and impair BRCA2 capability to protect MRE11-dependent degradation of stalled replication forks, leading to genomic instability and carcinogenic effects.

Recently, a novel carcinogenesis model in cells which are heterozygous for pathogenic *BRCA2* truncating mutations has been revealed by Tan et al. [29]; such cells should be capable of repairing DNA lesions using lower but still adequate levels of BRCA2 protein produced by the other intact copy of the gene, though that does not always happen. In their *in vitro* study, Tan et al. observed a selective dose-dependent proteasomal degradation of the remaining wild-type BRCA2 protein in the presence of naturally occurring concentrations of aldehydes, including formaldehyde and acetaldehyde. This selective depletion led to aldehyde-induced BRCA2 haploinsufficiency in cells with a heterozygous pathogenic *BRCA2* mutation, a situation in which critically low levels of the remaining BRCA2 protein are insufficient to perform normal cell functions [29]. Based on this observation, it is plausible to assume that *ALDH2* variants which lead to a lower activity of aldehyde dehydrogenase, the main enzyme to oxidize acetaldehyde to non-toxic acetate, may result in acetaldehyde accumulation and predispose cells with a *BRCA2* truncating mutation such as the p.K3326* mutation to the so-called aldehyde-induced BRCA2 haploinsufficiency and subsequent carcinogenic effects. One of those variants is *ALDH2* rs10744777 which is reported to be associated with the risk of ischemic stroke among European and East Asian populations [31,43]. Although the biological effect of this intronic variant on the protein product is not fully understood, it is regarded as an expression quantitative trait locus (eQTL) for *ALDH2* in monocytes, with the C allele associated with a higher expression level of the *ALDH2* compared to the T allele (beta = 0.159, p value = 8.75 E-11) [32]. It is possible that the presence of two T alleles of the *ALDH2* rs10744777 would affect gene expression level to the extent that would result in a non-optimal ALDH2 function and increased acetaldehyde build-up in cells. In the presence of the *BRCA2* p.K3326* mutation, non-detoxified accumulated acetaldehyde may induce BRCA2 haploinsufficiency by triggering selective degradation of the remaining BRCA2 protein which consequently makes cells prone to genomic instability and potential tumorigenesis. However, functional studies are needed to explicate the effect of the *ALDH2* rs10744777 on acetaldehyde detoxification and its potential contribution to aldehyde-induced BRCA2 haploinsufficiency.

Northeastern Iran has one of the highest reported rates of ESCC in the world. About three-fourths of incidental cases in this region is attributed to combined exposure to several risk factors, including thermal injuries, polycyclic aromatic hydrocarbons (from opium and indoor air pollution), nutrient deficiency, using un-piped water, and poor oral hygiene [8]. The ESCC declining trend in recent years is indicative of considerable improvements in local lifestyle and dietary habits [5,6]. Besides environmental exposure, genetic factors play a significant role in susceptibility to ESCC in this region [11,26,44]. The previous report by our team regarding the association of *BRCA2* p.K3326* mutation with ESCC risk [26] and our new finding of the considerable ESCC risk modifying effect of the *ALDH2*-rs10744777 TT genotype among *BRCA2* p.K3326* carriers signify the importance of determining genetically high-risk individuals for ESCC, so that we can address individualized preventive strategies and further help to reduce ESCC incidence in northeastern Iran. One important implication of our finding would be the impact of alcohol consumption on ESCC risk in individuals with co-existing TT genotype of the *ALDH2* rs10744777 and a germline *BRCA2* p.K3226* mutation. Although there is a well-documented role for alcohol consumption in susceptibility to esophageal cancer [45], such individuals are expected to be more vulnerable to ESCC risk due to the aldehyde-induced BRCA2 haploinsufficiency model. Therefore, these individuals may particularly benefit by limiting their alcohol consumption. Also, as another preventive strategy, they might benefit from dietary supplementations containing aldehyde scavengers such as Resveratrol [46]. Further research is needed to investigate the association between alcohol consumption and p.K3326*-associated ESCC risk in the setting of impaired aldehyde metabolism.

The major limitation of this study is the small sample size. However, considering that the cases were unselected and were recruited from major hospitals managing esophageal cancer patients in the studied regions and controls were mix of hospital- and population-based controls, we believe that our findings are generalizable to other populations. In particular, we have seen the same interaction on breast cancer risk in a much larger cohort among the Polish population [30].

In conclusion, we found that the *ALDH2*-rs10744777 TT genotype may be a significant risk modifier of ESCC in individuals with a *BRCA2* p.K3326* mutation. This observation may have implications for personalized primary preventive strategies to reduce ESCC incidence in northeastern Iran.

## Supporting information

S1 Checklist STROBE statement—checklist of items that should be included in reports of observational studies. (PDF)

S1 File Inclusivity in global research. (DOCX)
