# Supplementary material for: A Single nucleotide polymorphism in the ALDH2 gene modifies the risk of esophageal squamous cell carcinoma in BRCA2 p.K3326* carriers
Source: PLoS One. 2023 Nov 9;18(11):e0292611. doi: 10.1371/journal.pone.0292611 (PMC10635553; doi:10.1371/journal.pone.0292611)
Supplement: S1 Checklist — (PDF) [file pone.0292611.s001.pdf]

STROBE Statement—checklist of items that should be included in reports of observational studies

	Item No.	Recommendation	Page No.	Relevant text from manuscript
**Title and abstract**	1	(*a*) Indicate the study's design with a commonly used term in the title or the abstract	Page 1	**Background and Aims:** In the current case-control study, we aimed to investigate the ESCC risk modifying effect of this ALDH2 polymorphism among BRCA2 p.K3326* mutation carriers.
		(*b*) Provide in the abstract an informative and balanced summary of what was done and what was found	Page 1	**Methods:** We assessed the interaction between the ALDH2 rs10744777 polymorphism and BRCA2 p.K3326* mutation in ESCC risk by genotyping this ALDH2 variant in the germline DNA of 746 ESCC cases and 1,373 controls from northern Iran who were previously genotyped for the BRCA2 p.K3326* mutation. **Results:** Among a total of 464 individuals with TT genotype of the ALDH2 rs10744777 polymorphism, which is associated with lower ALDH2 gene expression, we found 9 of 164 cases versus 3 of 300 controls who carried the BRCA2 p.K3326* variant (OR = 5.66, 95% CI = 1.22–26.2, P = 0.018).
**Introduction**				
Background/rationale	2	Explain the scientific background and rationale for the investigation being reported Page 4		… Based on this observation (aldehyde-induced haploinsufficiency), it is plausible that mutations in *ALDH2*, a gene coding for aldehyde dehydrogenase to detoxify acetaldehyde, may elevate cancer risk in individuals with a heterozygous *BRCA2* truncating mutation such as the *BRCA2* p.K3326* mutation. To evaluate this hypothesis, our team recently investigated the association of the *BRCA2* p.K3326* mutation with breast cancer risk in the context of an *ALDH2* intronic variant (rs10744777) among 11,873 breast cancer patients and 7,615 ethnically matched controls from Poland [28]. The T allele of the *ALDH2* rs10744777 is associated with a lower expression level of the ALDH2 mRNA in blood lymphocytes compared to the C

				allele [29]. Among those who were homozygous for the *ALDH2*-rs10744777 T allele, the odds ratio (OR) for developing breast cancer associated with the *BRCA2* p.K3326* mutation was 1.72 (95% CI: 1.19-2.48, P = 0.003). While among those with CC/CT genotypes of the *ALDH2* rs10744777, the carriers of the *BRCA2* p.K3326* mutation did not have a higher risk of breast cancer compared to non-carriers (OR = 1.05, 95% CI: 0.73-1.51, P = 0.81). Our results suggest the breast cancer risk-modifying effect of the *ALDH2*-rs10744777 TT genotype among carriers of the *BRCA2* p.K3326* mutation.
Objectives	3	State specific objectives, including any prespecified hypotheses	Page 5	… Having observed this interaction in breast cancer, we aimed to investigate the association of the *BRCA2* p.K3326* mutation with ESCC risk in the context of the *ALDH2* rs10744777 variant.
**Methods**				
Study design	4	Present key elements of study design early in the paper		
Setting	5	Describe the setting, locations, and relevant dates, including periods of recruitment, exposure, follow-up, and data collection	Page 5	This study is a part of investigations into the etiology of upper gastrointestinal cancers in northern Iran [30]. Study subjects were from cities of Gonbad, the second largest city in Golestan province in northeastern Iran, and Ardabil, the largest city in Ardabil province in northwestern Iran, with high and intermediate rates of ESCC, respectively [31,32]. Cases and controls were recruited from August 2001 to May 2008.
Participants	6	(*a*) *Cohort study*—Give the eligibility criteria, and the sources and methods of selection of participants. Describe methods of follow-up Not Applicable<br>*Case-control study*—Give the eligibility criteria, and the sources and methods of case ascertainment and control selection. Give the rationale for the choice of cases and controls Pages 5 and 6<br>*Cross-sectional study*—Give the eligibility criteria, and the sources and methods of selection of participants Not Applicable		…Study subjects were from cities of Gonbad, the second largest city in Golestan province in northeastern Iran, and Ardabil, the largest city in Ardabil province in northwestern Iran, with high and intermediate rates of ESCC, respectively [31,32]. Cases and controls were recruited from August 2001 to May 2008. Verification of ESCC diagnosis for all cases was done by upper gastrointestinal endoscopy and subsequent evaluation of tumour biopsies…<br>…Control subjects were hospital patients with a health problem other than cancer (n = 898) or healthy individuals taken from the Golestan Cohort Study (GCS) in northeastern

			Iran (n = 475) [31]. No controls had a personal history of any type of cancer.<br>All available samples in our biobank that were previously tested for the *BRCA2* p.K3326* variant were tested for this study.
		(*b*) *Cohort study*—For matched studies, give matching criteria and number of exposed and unexposed Not Applicable<br>*Case-control study*—For matched studies, give matching criteria and the number of controls per case Not Applicable	
Variables	7	Clearly define all outcomes, exposures, predictors, potential confounders, and effect modifiers. Give diagnostic criteria, if applicable Clearly defined	
Data sources/ measurement	8*	For each variable of interest, give sources of data and details of methods of assessment (measurement). Describe comparability of assessment methods if there is more than one group Pages 6 and 7	The *BRCA2* p.K3326* variant was previously genotyped among all studied subjects using the TaqMan genotyping assay (*BRCA2* p.K3326* assay ID: C___27537307_20) on ABI 7500 fast real-time system (Applied Biosystems Co., Foster City, CA, USA). The *ALDH2* rs10744777 was genotyped among all study subjects by applying the TaqMan genotyping assay (*ALDH2* rs10744777 assay ID: C___2548076_10) on ABI 7500 fast real-time system (Thermo Fisher Scientific, Waltham, MA, USA).
Bias	9	Describe any efforts to address potential sources of bias Pages 6 and 7	There were 10% blinded duplicate samples in each plate; the mean concordance rate was 100%.
Study size	10	Explain how the study size was arrived at	All available samples in our biobank were tested for this study.

Continued on next page

Quantitative variables	11	Explain how quantitative variables were handled in the analyses. If applicable, describe which groupings were chosen and why Not Applicable		
Statistical methods	12	(*a*) Describe all statistical methods, including those used to control for confounding	Page 7	The permutation version of the exact test was done to test for Hardy-Weinberg Equilibrium. Fisher's exact test was applied to compare genotype frequencies between case and control subjects. Genotype comparisons were made under the dominant and recessive models. The significance level of α = 0.05 was used for all comparisons. We applied a multivariate logistic regression model to calculate adjusted odds ratios (ORs). Covariates included age, gender, ethnicity, smoking, alcohol drinking and opium use. Ethnicity was defined as Turkmen, Turk, Persian, Sistani, Balouch, or Kurd. All analyses were done by SNP & Variation Suite 8 (Golden Helix Inc., Bozeman, MT, USA).
		(*b*) Describe any methods used to examine subgroups and interactions		
		(*c*) Explain how missing data were addressed Not Applicable		
		(*d*) *Cohort study*—If applicable, explain how loss to follow-up was addressed Not Applicable<br>*Case-control study*—If applicable, explain how matching of cases and controls was addressed Not Applicable<br>*Cross-sectional study*—If applicable, describe analytical methods taking account of sampling strategy Not Applicable		
		(*e*) Describe any sensitivity analyses Not Applicable		
**Results**				
Participants	13*	(a) Report numbers of individuals at each stage of study—eg numbers potentially eligible, examined for eligibility, confirmed eligible, included in the study, completing follow-up, and analysed	Page 7	We studied 746 ESCC patients, including 380 (50.9%) males and 366 (49.1%) females, with the mean age of 63.6 years old (ranging from 25 to 89 years) at the time of diagnosis. The control group was composed of 1,373 individuals, including 700 (51.0%) males and 673 (49.0%) females, with the mean age of 55.2 years old (ranging from 24 to 90 years).

		(b) Give reasons for non-participation at each stage Not Applicable		
		(c) Consider use of a flow diagram Not Applicable		
Descriptive data	14*	(a) Give characteristics of study participants (eg demographic, clinical, social) and information on exposures and potential confounders	Page 16 (table 1)	
		(b) Indicate number of participants with missing data for each variable of interest Not Applicable		
		(c) *Cohort study*—Summarise follow-up time (eg, average and total amount) Not Applicable		
Outcome data	15*	*Cohort study*—Report numbers of outcome events or summary measures over time Not Applicable		
		Case-control study—Report numbers in each exposure category, or summary measures of exposure	Page 8	Of the total 464 individuals who had TT genotype of the *ALDH2* rs10744777, the frequency of the *BRCA2* p.K3326* variant was 5.49% (9 out of 164) among ESCC cases and 1.00% (3 out of 300) among controls (OR = 5.75, 95% CI = 1.53–21.5, *P* = 0.005). After adjusting for age, gender, ethnicity, smoking, alcohol drinking and opium use, *BRCA2* p.K3326* carriers had a 5.66-fold elevated risk of developing ESCC compared to non-carriers with TT genotype of the *ALDH2* rs10744777 (OR = 5.66, 95% CI = 1.22–26.2, *P* = 0.018).
		Cross-sectional study—Report numbers of outcome events or summary measures Not Applicable		
Main results	16	(*a*) Give unadjusted estimates and, if applicable, confounder-adjusted estimates and their precision (eg, 95% confidence interval). Make clear which confounders were adjusted for and why they were included	Page 8	Of the total 464 individuals who had TT genotype of the *ALDH2* rs10744777, the frequency of the *BRCA2* p.K3326* variant was 5.49% (9 out of 164) among ESCC cases and 1.00% (3 out of 300) among controls (OR = 5.75, 95% CI = 1.53–21.5, *P* = 0.005). After adjusting for age, gender, ethnicity, smoking, alcohol drinking and opium use, *BRCA2* p.K3326* carriers had a 5.66-fold elevated risk of developing ESCC compared to non-carriers with TT genotype of the *ALDH2* rs10744777 (OR = 5.66, 95% CI = 1.22–26.2, *P* = 0.018).
		(*b*) Report category boundaries when continuous variables were categorized Not Applicable		

	(*c*) If relevant, consider translating estimates of relative risk into absolute risk for a meaningful time period Not Applicable

Continued on next page

Other analyses	17	Report other analyses done—eg analyses of subgroups and interactions, and sensitivity analyses	Not Applicable	
**Discussion**				
Key results	18	Summarise key results with reference to study objectives	Page 8	The significant role of the *BRCA2* p.K3326* in susceptibility to ESCC among the studied population was previously reported by our team (OR = 3.64, 95% CI = 1.74-7.59, *P* = 0.0004) [24]. Here, we showed that among individuals who were homozygous for the *ALDH2* rs10744777 T allele, which is associated with a lower expression of *ALDH2*, the *BRCA2* p.K3326* mutation conferred a much higher risk of ESCC (OR = 5.66. 95% CI = 1.22-26.2, *P* = 0.018).
Limitations	19	Discuss limitations of the study, taking into account sources of potential bias or imprecision. Discuss both direction and magnitude of any potential bias	Page 11	The major limitation of our study is the small sample size, we cannot think of any specific bias in this study.
Interpretation	20	Give a cautious overall interpretation of results considering objectives, limitations, multiplicity of analyses, results from similar studies, and other relevant evidence	Pages 11 and 12	In conclusion, we found that the ALDH2-rs10744777 TT genotype may be a significant risk modifier of ESCC in individuals with a BRCA2 p.K3326* mutation. One important implication of our finding would be the impact of alcohol consumption on ESCC risk in individuals with co-existing TT genotype of the *ALDH2* rs10744777 and a germline *BRCA2* p.K3226* mutation. Although there is a well-documented role for alcohol consumption in susceptibility to esophageal cancer [41], such individuals are expected to be more vulnerable to ESCC risk due to the aldehyde-induced BRCA2 haploinsufficiency model. Therefore, these individuals may particularly benefit by limiting their alcohol consumption. Also, as another preventive strategy, they might benefit from dietary supplementations containing aldehyde scavengers such as Resveratrol [42]. Further research is needed to investigate the association between alcohol consumption and p.K3326*-associated ESCC risk in the setting of impaired aldehyde metabolism.
Generalisability	21	Discuss the generalisability (external validity) of the study results	Page 11	Considering that the cases are unselected and were recruited from major hospitals managing esophageal cancer cases in their regions and controls are mix of hospital and population controls, there is no reason to think that these results are not externally valid, in particular, since we have seen the same interaction on

			the risk of breast cancer in a much larger sample size in a completely different population (Poland).
**Other information**			
Funding	22	Give the source of funding and the role of the funders for the present study and, if applicable, for the original study on which the present article is based	This study was funded by Canadian Institutes of Health Research (CIHR). The founder had no role in the study other than funding it.

*Give information separately for cases and controls in case-control studies and, if applicable, for exposed and unexposed groups in cohort and cross-sectional studies.

Note: An Explanation and Elaboration article discusses each checklist item and gives methodological background and published examples of transparent reporting. The STROBE checklist is best used in conjunction with this article (freely available on the Web sites of PLoS Medicine at http://www.plosmedicine.org/, Annals of Internal Medicine at http://www.annals.org/, and Epidemiology at http://www.epidem.com/). Information on the STROBE Initiative is available at www.strobe-statement.org.
